# Colorimetric and fluorometric dual-mode determination of hypochlorite based on redox-mediated quenching†

**DOI:** 10.1039/d3ra05870k

**Published:** 2023-11-03

**Authors:** Ali O. AlQarni, Ashraf M. Mahmoud, Ramadan Ali, Mohamed M. El-Wekil

**Affiliations:** a Department of Pharmaceutical Chemistry, College of Pharmacy, Najran University Najran 11001 Saudi Arabia; b Department of Pharmaceutical Chemistry, Faculty of Pharmacy, University of Tabuk Tabuk 71491 Saudi Arabia r_ali@ut.edu.sa; c Department of Pharmaceutical Analytical Chemistry, Faculty of Pharmacy, Al Azhar University Assiut Branch 71526 Egypt; d Department of Pharmaceutical Analytical Chemistry, Faculty of Pharmacy, Assiut University Assiut 71526 Egypt mohamed.elwakeel@pharm.aun.edu.eg mohamed.mohamoud@ymail.com

## Abstract

We have successfully created a dual-modal probe, labeled as of iron(ii)–ortho-phenanthroline/N, S@g-CDs, which combines both fluorometric and colorimetric techniques for the accurate and sensitive detection of hypochlorite (ClO^−^). The mechanism behind this probe involves the fluorescence quenching interaction between nitrogen and sulfur co-doped green emissive carbon dots (N, S@g-CDs) and the iron(ii)–ortho-phenanthroline chelate, utilizing both the inner filter effect and redox processes. As the concentration of ClO^−^ increases, the iron(ii) undergo oxidation to iron(iii) as follows: Fe(ii) + 2HClO = Fe(iii) + Cl_2_O + H_2_O, leading to the restoration of N, S@g-CDs fluorescence. Simultaneously, the color of the system transitions gradually from red to colorless, enabling colorimetric measurements. In the fluorometric method with an excitation wavelength of 370 nm, the ClO^−^ concentration exhibits a wide linear correlation with fluorescence intensity ranging from 0.07 to 220 μM. The detection limit achieved in this method is 0.02 μM (S/N = 3). In contrast, the colorimetric method exhibits a linear range of 1.12 to 200 μM, with a detection limit of 0.335 μM (S/N = 3). The proposed selective absorbance for this method is 510 nm. The probe has been effectively utilized for the detection of ClO^−^ in various samples, including water and milk samples. This successful application showcases its potential for determining ClO^−^ in complex matrices, highlighting its broad range of practical uses.

## Abstract 2

We have successfully created a dual-modal probe, labeled as iron(ii)–ortho-phenanthroline/N, S@g-CDs, which combines both fluorometric and colorimetric techniques for the accurate and sensitive detection of hypochlorite (ClO^−^).

## Graphical abstract

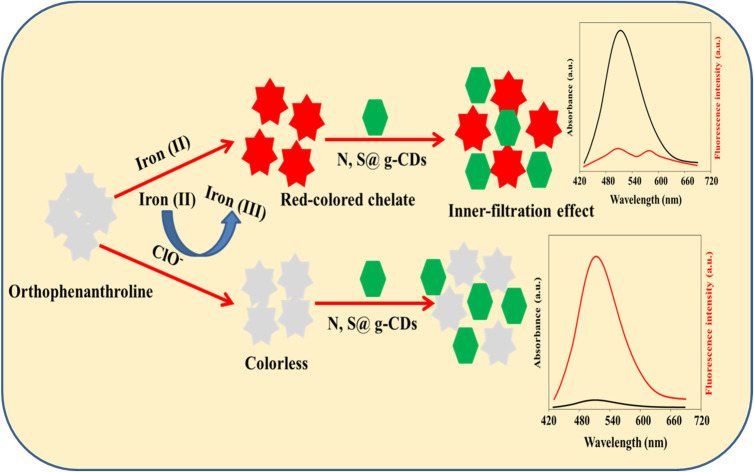

## 1. Introduction

Highlighting the significance of water safety is paramount, given its indispensable role in sustaining life. Water traverses a complex network of pumps, pipes, storage tanks, and distribution systems, creating potential opportunities for contamination along its journey.^1,2^ Over time, the quality of water tends to deteriorate, particularly in comparison to its source at the initial stages of the distribution network, thereby potentially endangering human health. To combat this, disinfection plays a pivotal role in water treatment, effectively eliminating or minimizing the presence of invisible yet harmful microorganisms. By doing so, it ensures the production of clean and safe water suitable for a wide range of applications. Moreover, the implementation of disinfection technologies serves as a preventive measure against the transmission of various diseases including, salmonellosis, typhoid, COVID-19, paratyphoid fevers, and shigellosis.^3,4^ The most common methods to disinfect water are physical and chemical methods. The latter involves chemical reagents such as ozone, silver, iodine, and chlorine. Chlorination is recognized as a highly effective public health measure, whereby chlorine, in the form of hypochlorite salts or Cl_2_ gas, is utilized for water disinfection.^5^ During chlorination processes, the primary reactive chlorine species is hypochlorous acid (HOCl). In the context of inorganic compounds, a rapid reaction between HOCl and substances such as ammonia, halides, SO_3_^2−^, CN^−^, NO_2_^−^, As(iii), and Fe(ii) has been documented, with reaction rates ranging from 10^3^ to 10^9^ M^−1^ s^−1^. On the other hand, lower reaction rates with Mn(ii) have been observed in homogeneous systems. Typically, the reactivity of chlorine arises from the initial electrophilic attack of HOCl on inorganic compounds.^6^ Moreover, ClO^−^ is added to milk as a sterilizing agent to increase its shelf-life.^7^ The excessive amount of ClO^−^ in water or milk produces potential health risks such as liver diseases,^8^ osteoarthritis,^9^ neuronal damage,^10^ cardiovascular problems,^11^ and carcinogenic effect.^12^ The limiting amount of Cl_2_ in water should not exceed 5.0 mg L^−1^, according to World Health Organization (WHO), to minimize its harmful effects in water.^13^ Therefore, it is imperative to construct an analytical method to estimate ClO^−^ in complicated biological and environmental systems selectively and sensitively. To date, many analytical techniques were used to quantify ClO^−^ such as potentiometry,^14^ ion-chromatography,^15^ colorimetry,^16,17^ chemiluminescence,^18^ and fluorometry.^19^ Despite the advantages of the colorimetric and fluorometric-based strategies for the detection of analytes, but they depend on a single-mode (rely solely on one quantitative technique), which influenced by environmental factors and non-standard assay procedures, affecting the reliability of the quantitation especially in complex matrices.^20,21^ Thus, it is desirable to construct multi-signal sensing mode for ClO^−^ detection with improved sensitivity, selectivity, and reliability.

Carbon dots (CDs) have received more interest in the area of chemical sensors because of high quantum fluorescence production, excellent water solubility, high stability, and ease of functionalization.^22–25^ The fluorescence emission wavelength of the as-prepared CDs is based on some conditions such as synthesis conditions, solvent used, and sources of synthesis.^26,27^ The existence of heteroatoms *e.g.* nitrogen (N) and sulfur (S) within the structure of CDs improves the internal properties of CDs such as optical properties, electronic properties, and surface chemical reactions.^28,29^ Hydrothermal method is the most common approach for preparing CDs as it easy, inexpensive, and allows for one pot synthesis for the heterogeneous reactions. It involves hydrolysis reaction under controlled temperature typically above 100 °C.^30,31^

Based on these facts, N, S@g-CDs was fabricated *via* a simple hydrothermal procedure, and designed as a dual-mode probe with iron(ii)–ortho-phenanthroline chelate for the ClO^−^ assay. The N, S@g-CDs probe emits fluorescence at 515 nm after excitation at 370 nm. The coordination interaction between iron(ii) and ortho-phenanthroline resulted in a red-colored chelate with an absorption band at 510 nm, which can quench the fluorescence of N, S@g-CDs *via* inner filtration effect (IFE). In the presence of ClO^−^, iron(ii) is oxidized to iron(iii) and the red-colored chelate is converted to colorless product. In addition, the emission of N, S@g-CDs was restored. As a result, a colorimetric and fluorometric bimodal nanoprobe was designed for determining ClO^−^ in different matrices.

## 2. Experimental

### 2.1. Materials and reagents

Cysteine (98.8%), alanine (97.6%), NaH_2_PO_4_ (AR), and Na_2_HPO_4_ (AR) were procured from Sigma-Aldrich. Sodium hypochlorite (NaOCl, AR), iron(ii) chloride (AR), 1,10-ortho-phenanthroline (AR), hydrochloric acid (HCl, AR), acetic acid (CH_3_COOH, AR), and sodium hydroxide (NaOH, AR) were procured from Merck.

### 2.2. Instruments

A SCINCO FluoroMate (FS-2, Korea) spectrofluorometer and Shimadzu 1601PC UV-vis scanning spectrophotometer were utilized to measure the fluorescence and absorption spectra, respectively. FTIR spectra were recorded using Nicolet™ iS™10 spectrometer. Philips X-ray diffractometer PW 1710 was used to record the PXRD pattern. TEM images were captured using a JEOL JEM-100CX II transmission electron microscope equipped with a tungsten EM filament with a voltage of 120 kV. DX analysis was performed with the NEX QC+ QuantEZ (Oxford, USA). The functional groups were identified using an X-ray photoelectron spectrometer (XPS, ESCA Ulvac-PHI 1600, USA).

### 2.3. Preparation of N, S@g-CDs

Hydrothermal method was used to prepare N, S@g-CDs. Briefly, 0.65 g of fine powdered banana peels biowaste, 0.35 g alanine, and 0.15 g cysteine were mixed with 25 mL deionized water, followed by sonication for 10 min. Following autoclaving at a temperature of 200 °C for duration of 10 h, the resultant mixture was allowed to cool to ambient temperature before being centrifuged for 20 min at 4000 rpm. Finally, it was purified using dialysis bags (MWCO = 3000 Da) for 24 h replacing the water every 6 h. The resultant solution was freeze-dried and stored in the refrigerator till use.

### 2.4. Steps for determination of hypochlorite (ClO^−^) anion

**Scheme 1:**
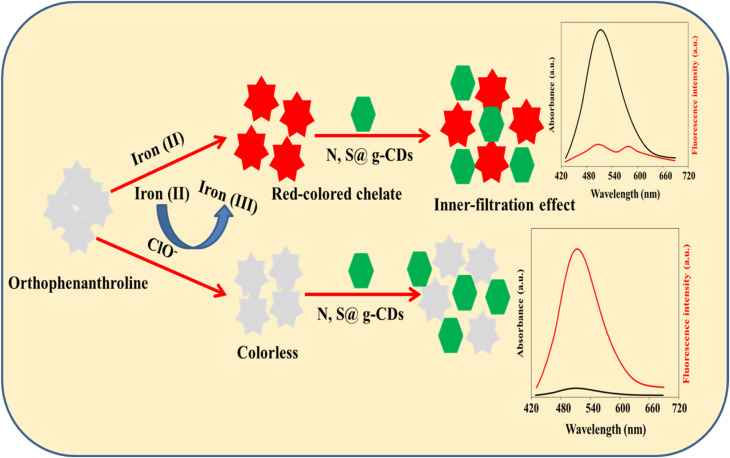
Representative diagram for the preparation of iron(ii)–ortho-phenanthroline/N, S@g-CDs system and its use for the quantitative determination of ClO^−^.

Fifty microliters of iron(ii) chloride (10.0 mM) was mixed with 350 μL of various amounts of ClO^−^ and 600 μL phosphate buffered solution (pH 6.0). Then, the above mixture was incubated at room temperature for 20 min. After that, 200 μL of 1,10-ortho-phenanthroline (10.0 mM) and 200 μL of 2.5 mg per mL N, S@g-CDs were added. The resultant solution was incubated at room temperature for 3 min before completing the volume to 2 mL with deionized water. Subsequently, the absorption spectra were captured at a wavelength of 510 nm, whereas the emission spectra were scanned at 515 nm following excitation at 370 nm (Scheme 1).

### 2.5. Sample preparation

Tap waters (pH = 7.08) were collected from our laboratories at Assiut University, Assiut, Egypt. Milk sample (5.0 mL) was mixed with 10 μL CH_3_COOH (to coagulate the colloids) and then subjected to centrifugation for 5 min at 10 000 rpm. The supernatant solution was collected and diluted with deionized water five times. The samples were analyzed before and following spiking with varying ClO^−^ amounts.

## 3. Results and discussions

### 3.1. Characterization

**Fig. 1:**
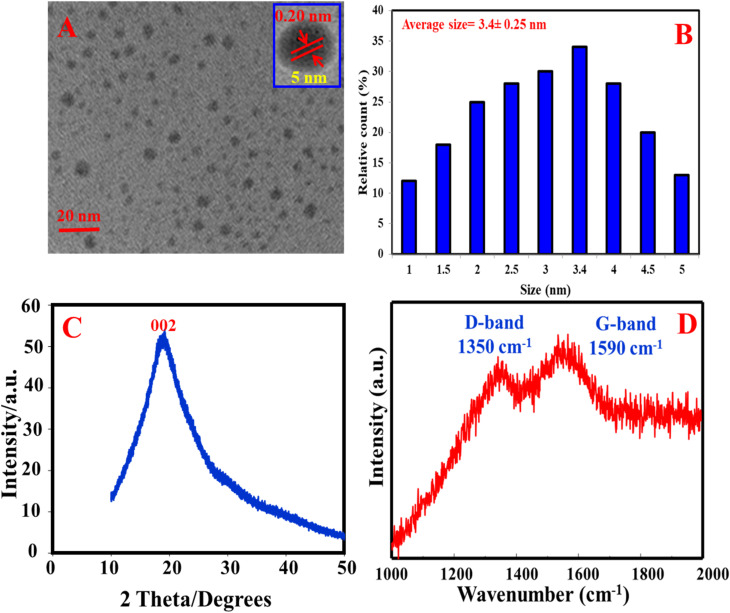
TEM (A), size distribution (B), PXRD (C), and Raman spectrum (D) of N, S@g-CDs.

**Fig. 2:**
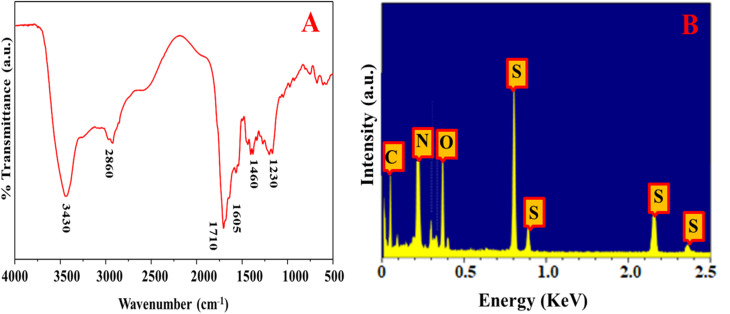
FTIR (A) and EDX (B) of N, S@g-CDs.

The morphology of the as-prepared N, S@g-CDs was investigated using TEM (Fig. 1A). It was found that the N, S@g-CDs probe has spherical shape with a uniform distribution. HRTEM (inset) reveals that the N, S@g-CDs exhibit high degree of crystallinity with a lattice spacing of 0.20 nm, affirming the (002) lattice fringes of graphene.^32^ The average particle size of N, S@g-CDs was found to be 3.4 nm ± 0.25 nm (Fig. 1B). Moreover, the PXRD pattern of the as-fabricated N, S@g-CDs was measured (Fig. 1C). It appears that the PXRD pattern of N, S@g-CDs exhibits a diffraction peak at 24.3°, which is consistent with the (002) plane of graphite. This suggests that the N, S@g-CDs have a graphene-like structure, but with a high degree of disorder.^27^ Raman spectrum of N, S@g-CDs explores two distinctive bands at 1350 cm^−1^ and 1590 cm^−1^ corresponding to the G- and D-bands of graphitic structure. The *I*_G_/*I*_D_ ratio was calculated as 1.06, affirming the graphitic-like structure of N, S@g-CDs as depicted in Fig. 1D.^33^ The FTIR spectrum of N, S@g-CDs was examined in Fig. 2A. The spectrum of N, S@g-CDs shows plenty of functional groups such as COOH, NH_2_, SH, and others. The presence of stretching vibrations at 3430, 2860, 1710, 1460, and 1230 cm^−1^ corresponds to OH/NH, CH_2_, C=O, C=C, and C–O–C/C–N–C, respectively.^34,35^ The EDX pattern of N, S@g-CDs was depicted in Fig. 2B where it was found prominent peaks of C, N, O, and S are present.

The XPS of the as-synthesized N, S@g-CDs was shown in Fig. S1A† with well-observed peaks of C 1s, N 1s, O 1s, and S 2p. Fig. S1B† exhibits the detailed XPS spectrum of C 1s with peaks positioned at 282.6 eV, 283.4 eV, and 284.8 eV, which assign to C=C/C–C, C–N, and C=O/C=N/C=S, respectively. Fig. S1C† shows the detailed XPS spectrum of N 1s with distinctive peaks at 397.7 eV and 398.9 eV, corresponding to pyridinic nitrogen and amidic/amino nitrogen, respectively. Fig. S1D† explores the detailed spectrum of O 1s with peaks at 528.8 eV and 529.5 eV, assigning to C=O and C–O–C/C–OH, respectively. Fig. S1E† exhibits two peaks that placed at 163.6 eV and 164.2 eV and correspond to S 2p_3/2_ and S 2p_1/2_, respectively.

**Fig. 3:**
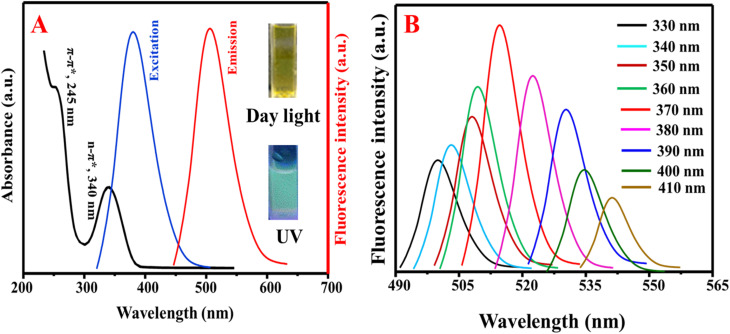
(A) The UV/vis and emission spectra of N, S@g-CDs and (B) dependency of emission wavelengths on the excitation wavelengths.

The optical properties of the as-fabricated N, S@g-CDs were investigated *via* UV/vis and fluorescence spectroscopic techniques (Fig. 3A). It was found that the N, S@g-CDs probe has two absorption bands at 245 nm and 340 nm, corresponding to π–π* (C=C) and n–π* (C=O, C=N) transitions, respectively.^36^ The fluorescence spectra of N, S@g-CDs exhibits fluorescence at 515 nm after excitation at 370 nm. Fig. 3B demonstrates that the fluorescence wavelengths depend on the excitation wavelengths where it is obvious that the emission wavelengths are red-shifted with fluorescence enhancement upon increasing the excitation wavelengths until optimum at 370 nm, and then gradually decreased. Thus, the excitation wavelength of 370 nm was selected as an optimum for subsequent determination. Stability of N, S@g-CDs was investigated in various conditions such as different pH, NaCl concentration, temperature, and irradiation times (Fig. S2†). It was found that the as-synthesized N, S@g-CDs probe unveils extreme high stability under different conditions, except for temperature where it was found that increasing the temperature decreases the fluorescence emission. Therefore, room temperature (25 °C) was chosen as a suitable temperature for N, S@g-CDs.

### 3.2. Sensing mechanism, optimization of variables, and detection feasibility

In order to verify the mechanism of ClO^−^ sensing using the as-prepared nanosystem, the absorption spectrum of iron(ii)–ortho-phenanthroline and emission spectrum of N, S@g-CDs were recorded in Fig. S3A.† It was found that the iron(ii)–ortho-phenanthroline complex has an absorption band at *λ*_max_ of 510 nm, while the emission of N, S@g-CDs at 515 nm. The overlapping between the absorption spectrum of iron(ii)–ortho-phenanthroline complex and fluorescence spectrum N, S@g-CDs is responsible for the quenching of N, S@g-CDs' fluorescence emission through inner-filtration effect (IFE). To further prove that the mechanism is IFE, fluorescence lifetimes of N, S@g-CDs were measured before and following addition of iron(ii)–ortho-phenanthroline complex (Fig. S3B†). It was found that the emission lifetimes of N, S@g-CDs before and following adding of N, S@g-CDs are 5.34 ns and 5.29 ns, proving the fluorescence lifetimes were not appreciably changed. The data obtained from fluorescence lifetimes proved that the mechanism of the interaction between N, S@g-CDs and iron(ii)–ortho-phenanthroline complex is IFE.^37^ I t is noteworthy mentioning that the ortho-phenanthroline can form stable red-colored chelate with iron(ii) in pH range of 3.0 to 10.0. Moreover, different metal cations were investigated for their chelation with ortho-phenanthroline including sodium (Na^+^), potassium (K^+^), calcium (Ca^2+^), magnesium (Mg^2+^), zinc (Zn^2+^), copper (Cu^2+^), cobalt (Co^2+^), ferric (Fe^3+^), and ferrous (iron ii, Fe^2+^). The data depicted in Fig. S3C† confirms that only iron(ii) can form strong chelate with strong absorption, which can significantly quench the fluorescence emission of N, S@g-CDs. In addition, the molar ratio of iron(ii) : ortho-phenanthroline was demonstrated in Fig. S3D.† It was found that the maximum fluorescence quenching was obtained with 1 : 4, after that the fluorescence responses became steady and did not appreciably change. Fig. S3E† demonstrates that the increase in iron(ii) concentration decreased the emission of N, S@g-CDs. Consequently, 250 μM iron(ii) and 1000 μM ortho-phenanthroline were used for subsequent studies.

Fluorescence and absorption spectra were scanned following to the addition of N, S@g-CDs, ortho-phenanthroline/N, S@g-CDs, iron(ii) : ortho-phenanthroline/N, S@g-CDs, and iron(ii) + ClO^−^ + ortho-phenanthroline/N, S@g-CDs in order to demonstrate the viability of implementing a fluorometric and colorimetric technique for quantifying ClO^−^. Fig. S4A† exhibits the emission spectrum of N, S@g-CDs (curve a), which was not changed after addition of ortho-phenanthroline (curve b). Addition of iron(ii) to ortho-phenanthroline/N, S@g-CDs (curve c) quenched the emission of N, S@g-CDs with emerging a new band at 575 nm, which may be an interaction product between N, S@g-CDs and iron(ii) : ortho-phenanthroline chelate (Fig. S4B†). Addition of ClO^−^ recovered the fluorescence emission of N, S@g-CDs as a result oxidation of iron(ii) to iron(iii), (curve d). Fig. S4C† shows the UV/vis of N, S@g-CDs, ortho-phenanthroline/N, S@g-CDs, iron(ii) : ortho-phenanthroline/N, S@g-CDs, and iron(ii) + ClO^−^ + ortho-phenanthroline/N, S@g-CDs. It was found that N, S@g-CDs has no absorption at 510 nm (curve a). Addition of ortho-phenanthroline to N, S@g-CDs did not improve the absorption intensity (curve b). For iron(ii) : ortho-phenanthroline/N, S@g-CDs, the absorption intensity at 510 nm was significantly increased as a result of formation of red-colored chelate of iron(ii)–ortho-phenanthroline (curve c). In the presence of ClO^−^, the absorption intensity at 510 nm was dramatically decreased as a result of oxidation of iron(ii) to iron(iii). Fig. S4D† exhibits the UV/vis spectra of ortho-phenanthroline after addition of iron(ii), iron(iii), and ClO^−^. It was found that the intense absorption was obtained with iron(ii) : ortho-phenanthroline, which was decreased after addition of ClO^−^. The influence of the pH and incubation time on the dual-mode detection of ClO^−^ was demonstrated (Fig. S5†). Fig. S5A† explores the influence of the pH value on the absorbance of 50 μM ClO^−^. It was found that the absorbance values were increased with the pH until the optimum value was obtained at pH 6.0, and then gradually decreased. This may be attributed to the high oxidation power of ClO^−^ that could be obtained in weak acidic medium.^38^ Moreover, at low pH values the complexation between iron(ii) and ortho-phenanthroline would slow down. The reaction between ClO^−^ and Fe(ii) can be described as follows: Fe(ii) + 2HClO = Fe(iii) + Cl_2_O + H_2_O. Fig. S5B† shows the influence of the incubation time after the addition of ClO^−^. It was found the optimum ratio was obtained after 20 min. Therefore, 20 min was selected as an ideal reaction time for the reaction of ClO^−^.

### 3.3. Dual-mode detection of ClO^−^

**Fig. 4:**
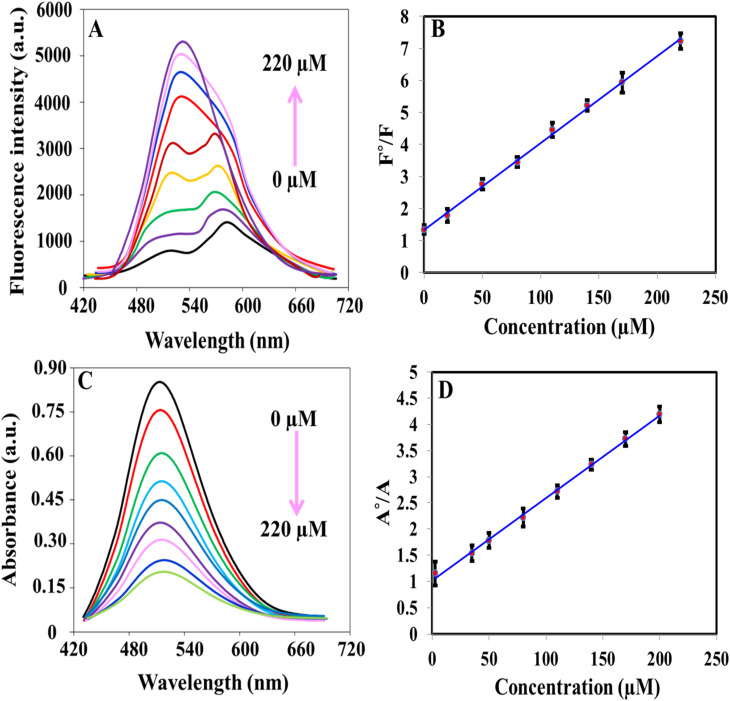
(A) Fluorescence spectra of iron(ii)–ortho-phenanthroline/N, S@g-CDs system in the presence of different concentrations of ClO^−^ (0–220 μM); (B) calibration plot of *F*°/*F versus* concentration of ClO^−^; (C) absorption spectra of iron(ii)–ortho-phenanthroline/N, S@g-CDs system in the presence of different concentrations of ClO^−^ (0–200 μM); (D) calibration plot of *A*°/*A versus* concentration of ClO^−^.

The iron(ii)–ortho-phenanthroline/N, S@g-CDs system was used for the determination of ClO^−^ colorimetrically and fluorometrically (Fig. 4). Fig. 4A shows that the fluorescence emission of the system was increased with increasing the concentration of ClO^−^ in the range of 0.07–220 μM. Fig. 4B exhibits the calibration plot between *F*°/*F* (*F*° and *F* refer to the fluorescence before and after addition of ClO^−^) and ClO^−^ concentration. The linear regression equation can be described as *F*°/*F* = 1.293 + 0.0275*C*_ClO^−^_ while the detection limit (LOD) is calculated as 0.02 μM according to S/N = 3. Fig. 4C demonstrates the absorption spectra of iron(ii)–ortho-phenanthroline/N, S@g-CDs system in the presence of different concentrations of ClO^−^ (1.12–200 μM). The linear regression can be written as *A*°/*A* = 1.008 + 0.0358*C*_ClO^−^_ where *A*° and *A* refer to the absorbance before and after addition of ClO^−^. The detection limit (LOD) of the colorimetric method was calculated as 0.335 μM (S/N = 3), Fig. 4D. The analytical performance of the as-prepared system was compared with other methods (sensors) devoted for the determination of ClO^−^ (Table 1). It was concluded that the proposed system exhibits many advantages over existing methods including wide-dynamic linear range, low detection limit, and detection reliability.

**Table 1:** Comparison between analytical parameters of the presented system and other methods (sensors) for the detection of ClO^−^a

Method	System	Linear range (μM)	LOD (μM)	Reference
Colorimetry	Sequential injection/TMB	1.76–254.9	1.57	16
	AuNSs@AgNRs	0.5–30	0.24	17
	Merocyanine dye	0–24	0.42	39
	G-CDs	10–150	1.82	40
	BN-CDs	300–4000	95.7	41
	**Iron(** **ii** **)–ortho-phenanthroline/N, S@g-CDs**	**1.12–200**	**0.335**	**This work**
Fluorometry	G-CDs	0.2–100	0.0781	40
	BN-CDs	0.1–1000	0.045	41
	N/B-CNPs	5–50	0.12	42
	CDs	0.5–50	0.23	43
	Bicyclic 2-pyridone-based fluorescent probe	0–85	1.32	44
	**Iron(** **ii** **)–ortho-phenanthroline/N, S@g-CDs**	**0.07–220**	**0.02**	**This work**

a TMB: tetramethylbenzidine; NSs: nanospheres; NRs: nanorods; G-CDs; green carbon dots; BN-CDs: boron and nitrogen co-doped carbon dots; CDs: carbon dots.

### 3.4. Selectivity

**Fig. 5:**
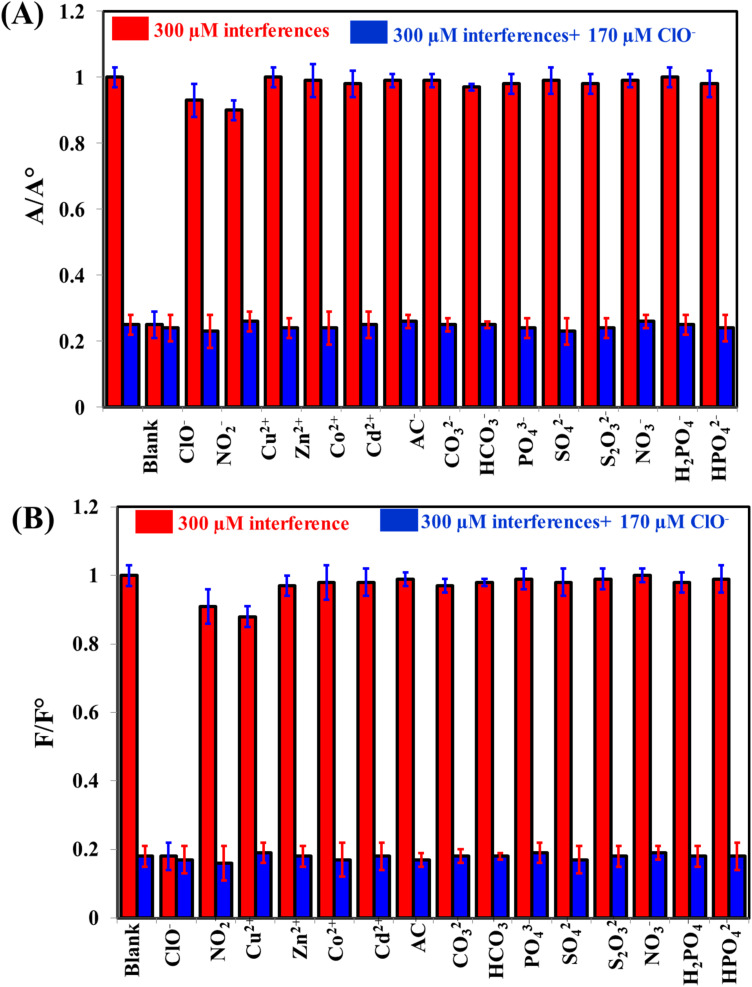
The selectivity of iron(ii)–ortho-phenanthroline/N, S@g-CDs for the detection of 170 μM ClO^−^ in the presence of 300 μM interfering species either colorimetrically (A) or fluorometrically (B).

To evaluate the selectivity of the system towards the measurement of ClO^−^, common interfering cations and anions were tested such as NO_2_^−^, Cu^2+^, Zn^2+^, Co^2+^, Cd^2+^, AC^−^, CO_3_^2−^, HCO_3_^−^, PO_4_^3−^, SO_4_^2−^, S_2_O_3_^2−^, NO_3_^−^, H_2_PO_4_^−^, and HPO_4_^2−^ (Fig. 5). It is clearly seen that the absorbance readings of as-prepared system can be decreased by ClO^−^ (Fig. 5A), while the fluorescence readings can be recovered by ClO^−^ (Fig. 5B). These results can confirm the selectivity of the system towards by ClO^−^ detection.

### 3.5. Applications

**Fig. 6:**
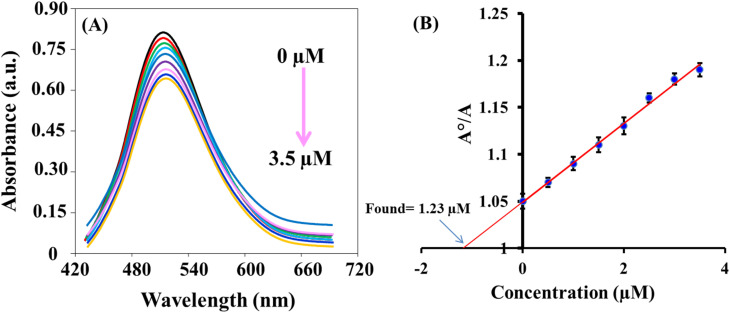
(A) Absorption spectra of iron(ii)–ortho-phenanthroline/N, S@g-CDs system in water sample and in the presence of different concentrations of ClO^−^ (0–3.5 μM); (B) the standard addition plot for the determination between *A*°/*A versus* the concentration of ClO^−^.

The sensing platform that was developed successfully detected ClO^−^ in tap water and milk samples. The results, as shown in Table 2, demonstrated a recovery rate of 95.7–105.7%. These findings indicate the accuracy and reliability of the dual-mode based sensing system. Additionally, the concentrations of the spiked samples were also measured using the *N*,*N*-diethyl-*p*-phenylenediamine (DPD) colorimetric method,^45^ which yielded results that were consistent with the colorimetric and fluorescence methods. This demonstration highlights the potential applications of this fast and reliable system. Fig. 6 exhibits the standard addition plot for the determination of ClO^−^ in water sample using colorimetric method.

**Table 2:** Determination of ClO^−^ in tap water and milk samples using the presented and reported methods (*n* = 5)

Samples	Added (μM)	Colorimetric method			Fluorometric method			Reported method^45^		
		Found (μM)	Recovery%	RSD%	Found (μM)	Recovery%	RSD%	Found (μM)	Recovery%	RSD%
Tap water	0.0	1.23	—	—	1.15	—	—	1.19	—	—
	0.5	1.83	105.7	2.8	1.63	98.8	3.2	1.62	95.9	3.9
	1.0	2.18	97.8	3.2	2.12	98.6	3.1	2.08	94.9	4.2
	2.0	3.20	99.1	3.4	3.09	95.7	2.5	3.22	100.9	3.6
Milk	0.0	ND	—	—	ND	—	—	ND	—	—
	0.5	0.48	96.0	3.3	0.52	104.0	3.2	0.53	106.0	3.5
	1.0	1.04	104.0	3.1	1.01	101.0	3.3	1.05	105.0	4.3
	2.0	1.98	99.0	2.8	1.97	98.5	3.2	2.05	102.5	3.8

## 4. Conclusion(s)

In summary, we have developed a dual-modal nanoprobe, combining fluorometric and colorimetric methods, for the sensitive and selective detection of hypochlorite (ClO^−^) anion. The nanoprobe exploits the inner filter effect (IFE) and redox reactions. By coordinating ortho-phenanthroline with iron(ii), a red chelate was formed, which effectively quenches the fluorescence of N, S@g-CDs through IFE. Upon the introduction of ClO^−^, iron(ii) undergoes oxidation to iron(iii), resulting in the formation of a colorless chelate and the restoration of the fluorescence of N, S@g-CDs. This approach enables both colorimetric and fluorometric determination of ClO^−^. As a result, this nanoprobe can be applied for the detection of ClO^−^ in tap water and milk samples and holds potential for on-site ClO^−^ testing.

## Conflicts of interest

The authors declare that they have no known competing financial interests or personal relationships that could have appeared to influence the work reported in this paper.

## Supplementary Material

RA-013-D3RA05870K-s001
