# Supplementary material for: Colorimetric and fluorometric dual-mode determination of hypochlorite based on redox-mediated quenching
Source: RSC Adv. 2023 Nov 3;13(46):32492–501. doi: 10.1039/d3ra05870k (PMC10624236; doi:10.1039/d3ra05870k)
Supplement: RA-013-D3RA05870K-s001 [file RA-013-D3RA05870K-s001.pdf]

Electronic Supplementary Material (ESI) for RSC Advances.
This journal is © The Royal Society of Chemistry 2023

Electronic Supplementary Material

Colorimetric and fluorometric dual-mode determination of hypochlorite based on redox-mediated quenching

Ali O. AlQarni[a], Ashraf M. Mahmoud[a], Ramadan Ali[b,c*], Mohamed, M. El-Wekil[d*]

[a] Department of Pharmaceutical Chemistry, College of Pharmacy, Najran University, Najran, 11001, Saudi Arabia

[b] Department of Pharmaceutical Chemistry, Faculty of Pharmacy, University of Tabuk, Tabuk71491, Saudi Arabia

[c] Department of Pharmaceutical Analytical Chemistry, Faculty of Pharmacy, Al Azhar University, Assiut Branch, 71526, Egypt

[d] Department of Pharmaceutical Analytical Chemistry, Faculty of Pharmacy, Assiut University, Assiut, 71526, Egypt

*Ramadan Ali: r_ali@ut.edu.sa

*Mohamed M. El-Wekil: mohamed.elwakeel@pharm.aun.edu.eg, mohamed.mohamoud@ymail.com

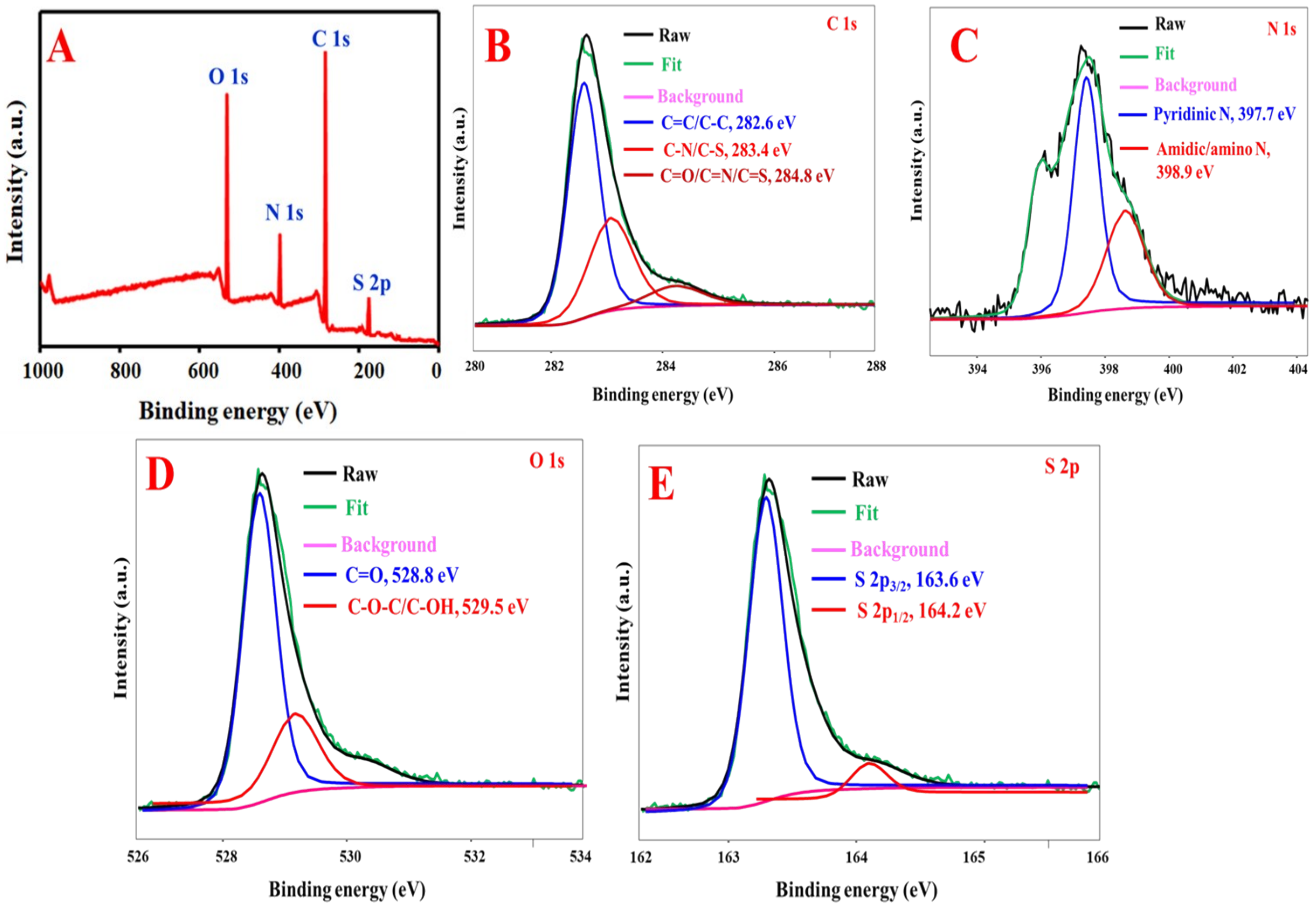

Fig.S1 (A) Full XPS spectrum of N, S@ g-CDs while (B), (C), (D), and (E) represents detailed XPS of C 1s, N 1s, O 1s, and S 2p, respectively.

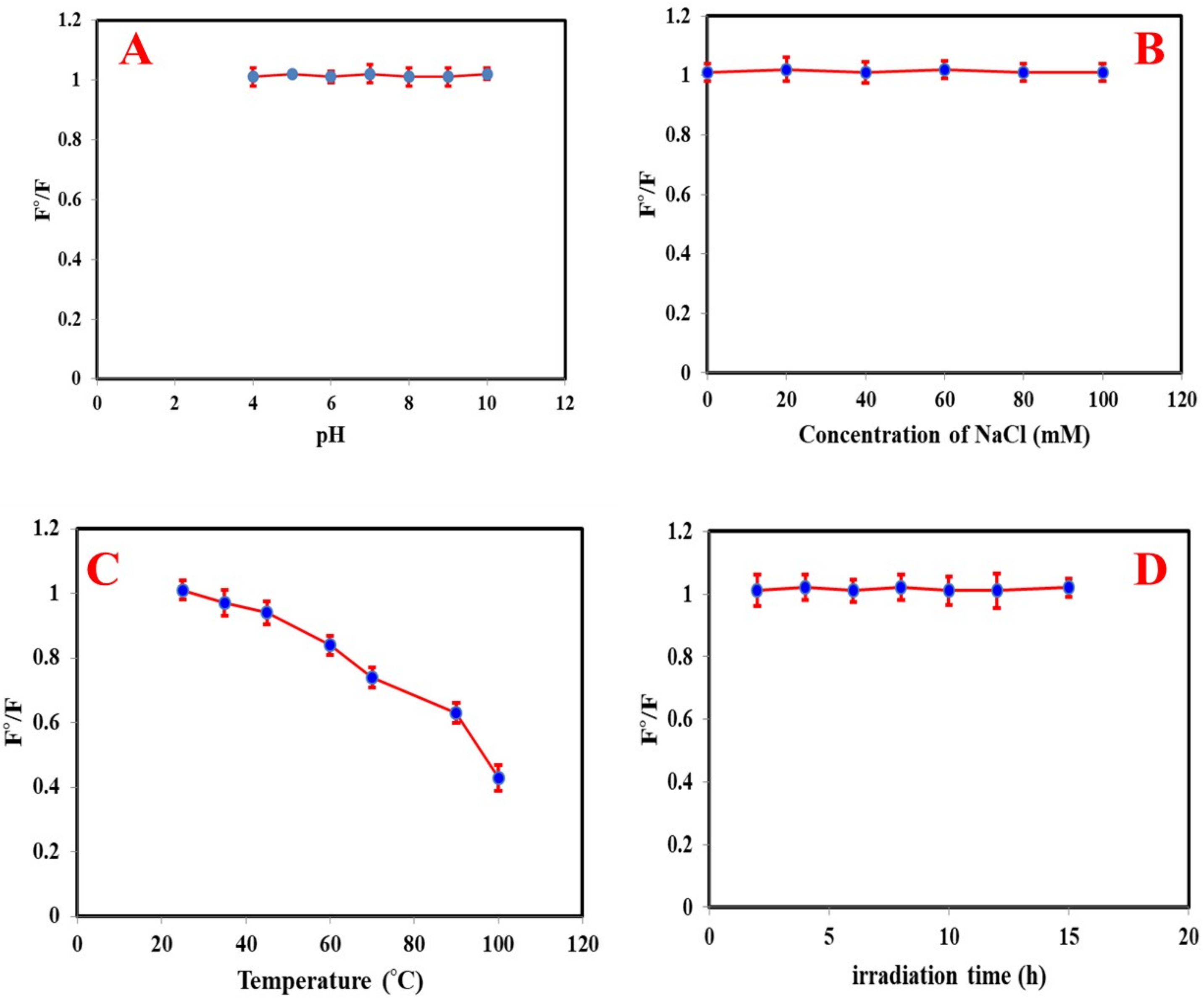

Fig.S2 The effect of pH (A), concentration of NaCl (B), temperature (C), and irradiation time (D) on the stability of N, S@ g-CDs.

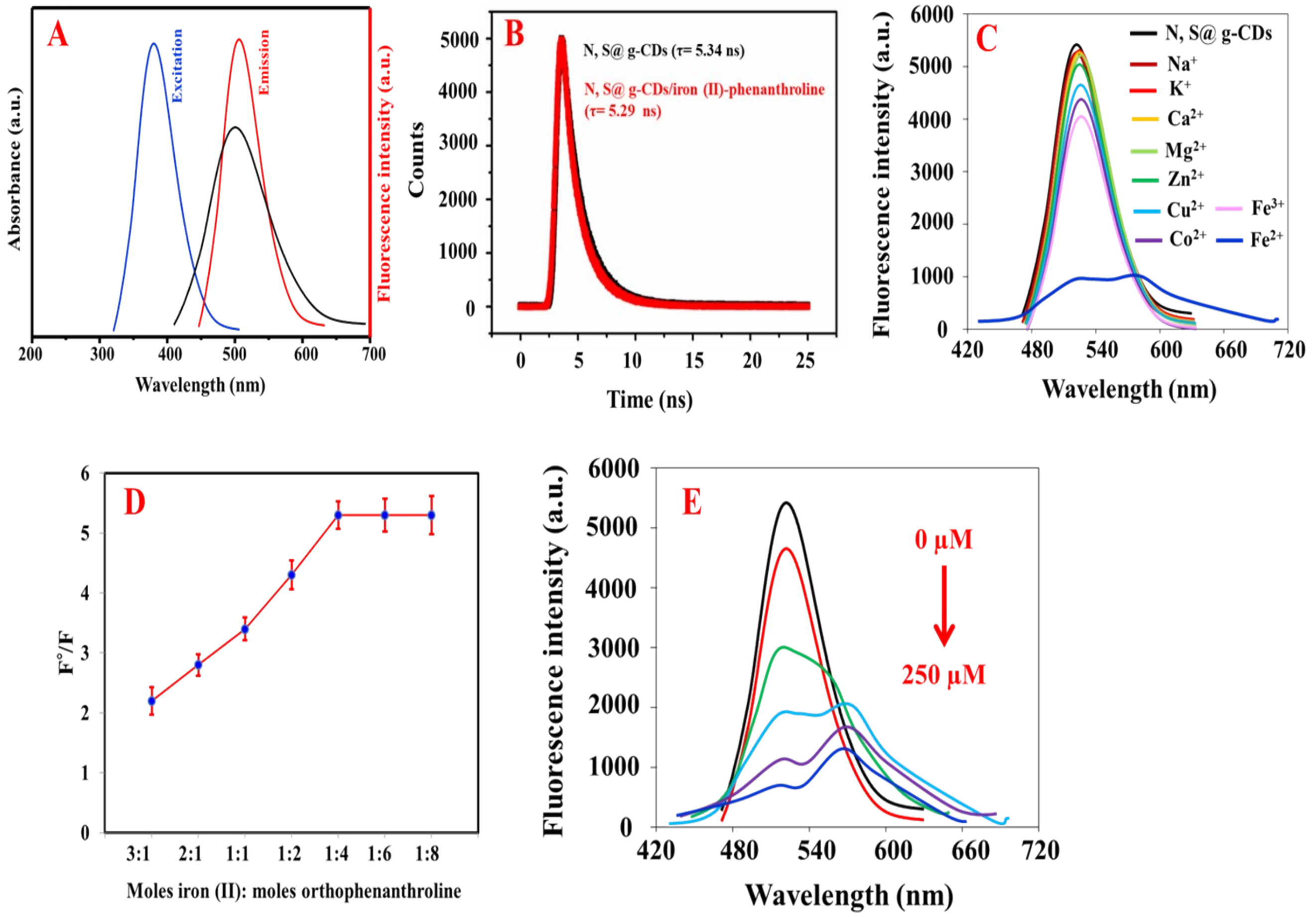

Fig.S3 (A) Absorption spectrum (black line) of iron (II)-orthophenanthroline chelate and excitation/emission spectra of N, S@ g-CDs; (B) Fluorescence lifetimes of N, S@ g-CDs before and after addition of iron (II): orthophenenthroline; (C) Specificity of orthophenanthroline towards different metal cations; (D) Effect of iron (II): orthophenanthroline ratios on the fluorescence responses of N, S@ g-CDs; (E) Effect of iron (II) concentrations on the fluorescence responses of N, S@ g-CDs.

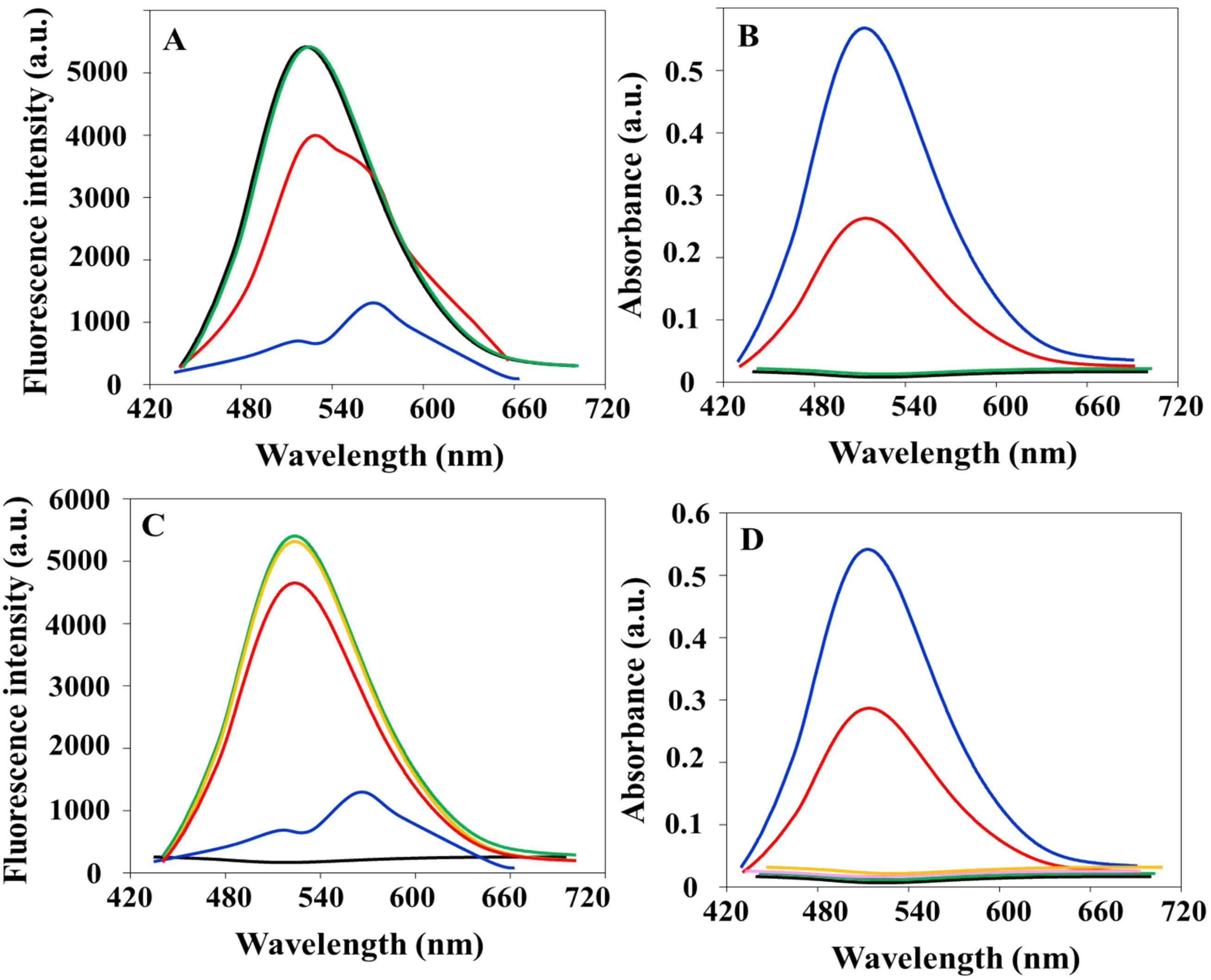

Fig.S4 (A) Fluorescence spectra of N, S@ g-CDs (black), orthophenanthroline/ N, S@ g-CDs (green), iron (II)- orthophenenthroline/ N, S@ g-CDs (blue), iron (II)+ ClO^{-}+orthophenenthroline/ N, S@ g-CDs (red); (B) Fluorescence spectra of iron (II)- orthophenenthroline (black), N, S@ g-CDs (green), N, S@ g-CDs/ orthophenanthroline (orange), iron (II)/ N, S@ g-CDs (red), iron (II)- orthophenenthroline/ N, S@ g-CDs (blue); (C) Absorption spectra of N, S@ g-CDs (black), orthophenanthroline/ N, S@ g-CDs (green), iron (II)- orthophenenthroline/ N, S@ g-CDs (blue), iron (II)+ ClO^{-}+orthophenenthroline/ N, S@ g-CDs (red); (D) Absorption spectra of orthophenenthroline (black), iron (III)- orthophenenthroline (green), iron (II)- orthophenenthroline (blue), iron (II)+ ClO^{-}+orthophenenthroline (red), iron (III)+ ClO^{-}+orthophenenthroline (orange).

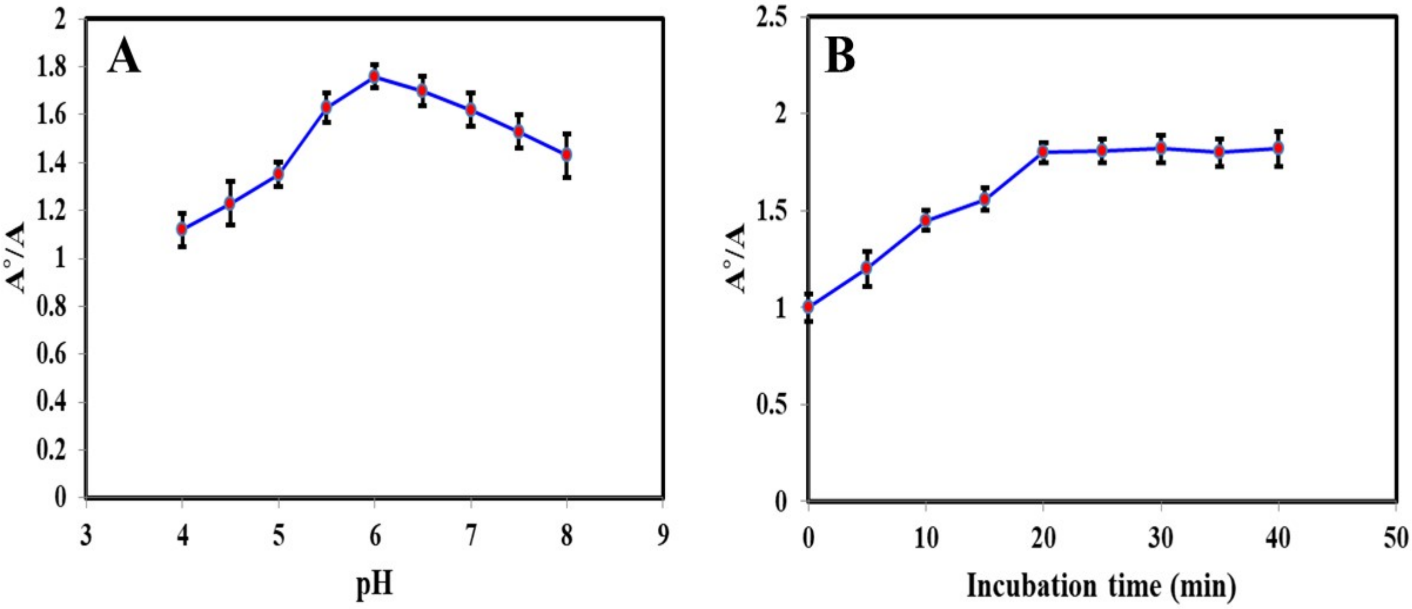

Fig.S5 The effect of pH (A) and incubation time (B) on the colorimetric determination of 50 μM ClO^- anion.
